# GPU-Based Block-Wise Nonlocal Means Denoising for 3D Ultrasound Images

**DOI:** 10.1155/2013/921303

**Published:** 2013-11-03

**Authors:** Liu Li, Wenguang Hou, Xuming Zhang, Mingyue Ding

**Affiliations:** Department of Biomedical Engineering, School of Life Science and Technology, Key Laboratory of Image Processing and Intelligence Control of Education Ministry of China, Huazhong University of Science and Technology, No. 1037, Luoyu Road, Wuhan 430074, China

## Abstract

Speckle suppression plays an important role in improving ultrasound (US) image quality. While lots of algorithms have been proposed for 2D US image denoising with remarkable filtering quality, there is relatively less work done on 3D ultrasound speckle suppression, where the whole volume data rather than just one frame needs to be considered. Then, the most crucial problem with 3D US denoising is that the computational complexity increases tremendously. The nonlocal means (NLM) provides an effective method for speckle suppression in US images. In this paper, a programmable graphic-processor-unit- (GPU-) based fast NLM filter is proposed for 3D ultrasound speckle reduction. A Gamma distribution noise model, which is able to reliably capture image statistics for Log-compressed ultrasound images, was used for the 3D block-wise NLM filter on basis of Bayesian framework. The most significant aspect of our method was the adopting of powerful data-parallel computing capability of GPU to improve the overall efficiency. Experimental results demonstrate that the proposed method can enormously accelerate the algorithm.

## 1. Introduction

Ultrasonic imaging owns advantages such as noninvasive, radiation-free, low-cost, and fast imaging compared with other medical imaging techniques [1]. It has been widely used in many medical applications. Since 3D ultrasound imaging can provide clearer spatial relationship and more abundant diagnostic information compared with 2D ultrasound, it attracts much attention from the related fields. However, due to the coherence properties of ultrasound imaging, the image is often severely corrupted by speckle and other artifacts. Speckle could obscure the important image details and reduce the contrast of the soft tissues in the image, thereby causing great difficulties to the subsequent US image processing such as edge detection, image segmentation, and image registration. Therefore, an efficient 3D ultrasound image denoising algorithm is in urgent need in the field of 3D ultrasound.

Many researchers engaged in image processing have proposed lots of denoising algorithms for 2D ultrasound images [1–3]. However, only a few methods were presented for 3D ultrasound speckle suppression. Yue and Clark [4] introduced a speckle suppression approach by an integration of the 3D nonlinear diffusion and 3D dyadic wavelet transform techniques, in which, normalized wavelet modulus was used as an edge map to expose the intrinsic speckle/edge relation. Based on a local distribution of variance for a given voxel, Veronika et al. [5] presented a structure-preserving filter specifically designed to eliminate the speckle and random noise in 3D ultrasound datasets. Coupé et al. [6] proposed a modified Bayesian nonlocal means algorithm deduced from a relevant ultrasound noise model to accurately preserve edges and structural details of the image.

The basic idea of the nonlocal means [7] method is that the image contains a large number of repeat modes, and they can be utilized to reduce the random noises by averaging operation. Despite the superiority of the NLM algorithm in preserving image details, it involves high computational complexity. While applying the NLM algorithm to 3D image denoising, the computational burden is especially huge since the algorithm needs to take into account the relevant information in all three dimensions. It will lead to a relatively long runtime for general CPUs, which hinders the employment of the algorithm in practical medical applications.

Three strategies can be used for the algorithm acceleration: the multithread CPU technology with multicore CPU, the multi CPU technology based on high-performance computer clusters or servers, and the GPU technology [8]. Though multicore CPU and multithread technology can be used to accelerate the algorithm, the maximum ratio of speedup is approximately equivalent to the number of CPU cores. Besides, the coarse multithread of CPU is in software level, which is time costly when switching among different threads. While the high-performance computer clusters can improve the processing speed very much, the high cost of owning and maintaining makes them difficult to access for most researchers and clinical users [8]. Comparatively speaking, the GPU can get a good balance between the cost and performance. The GPU adopts the light level threads of hardware management, so the overhead of threads switching can achieve zero. For example, when a thread is waiting for addressing off-chip memory or synchronic commands, the GPU can rapidly switch to another thread on deck, thus hiding the latency by calculation. Besides, as a highly parallel, multithread and multicore processor, GPU can provide tremendous computational horsepower and very high memory bandwidth. Therefore, it is very good at addressing such problems that can be expressed as data-parallel computations—the execution of the same program on many data elements in parallel—with high arithmetic intensity. 

Compute unified device architecture (CUDA), officially released by NVIDIA Corporation in 2007, comes with a software environment in which the developers can use C-like language rather than computer graphics API for general purpose computing of GPU (GPGPU). Many applications that process large data sets have used the CUDA programming model to speed up the computation [9–12]. Some examples in medical image processing that have taken advantages of the computational power of the GPU are image registration [13], image segmentation [14], f-MRI analysis [15], and so on. In the field of image denoising, some researchers have tried to employ GPU to accelerate 2D image denoising. In 2007, Chen et al. [16] implemented bilateral filtering on GPU for real-time edge-aware image processing. Su and Xu [17] proposed how to accelerate wavelet-based image denoising by GPU. Fontes et al. [18] adopted the GPU for real-time denoising of 2D ultrasound data. Goossens et al. [19] managed to run the commonly used nonlocal means algorithm in real time. In this paper, we intend to use the great computational power of GPU to implement the Bayesian block-wise NLM filter to realize fast 3D ultrasound speckle reduction on basis of Coupé's work.

## 2. Method

### 2.1. GPU and CUDA

**Figure 1:**
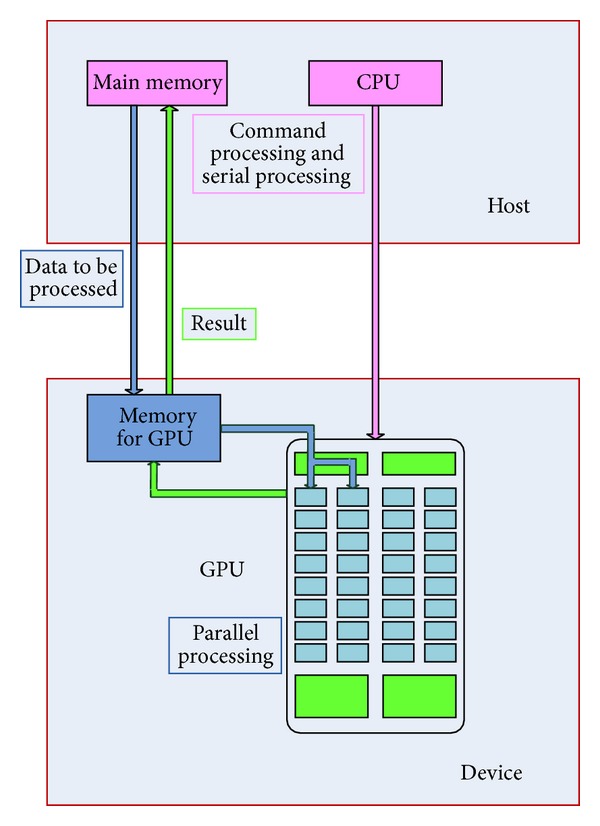
Programming model of CUDA.

In this section, we will briefly introduce the structure of CUDA, in which three important concepts are involved: host, device, and kernel. In CUDA programming model, shown as Figure 1, CPU is considered as the host to responsible for logical transaction processing and serial computing while GPU serves as the device to focus on the implementation of parallel processing tasks. They work together as a complete model and perform their own duties within the model. Once the parallel portion of the program is determined, we can hand the computation task of this part over to GPU. Function of CUDA parallel computing running on the GPU is called kernel, which is not a complete program but just a step that can be executed in parallel. A complete CUDA program is composed of a series of parallel procedures of kernel functions on device and serial procedures on host. 

**Figure 2:**
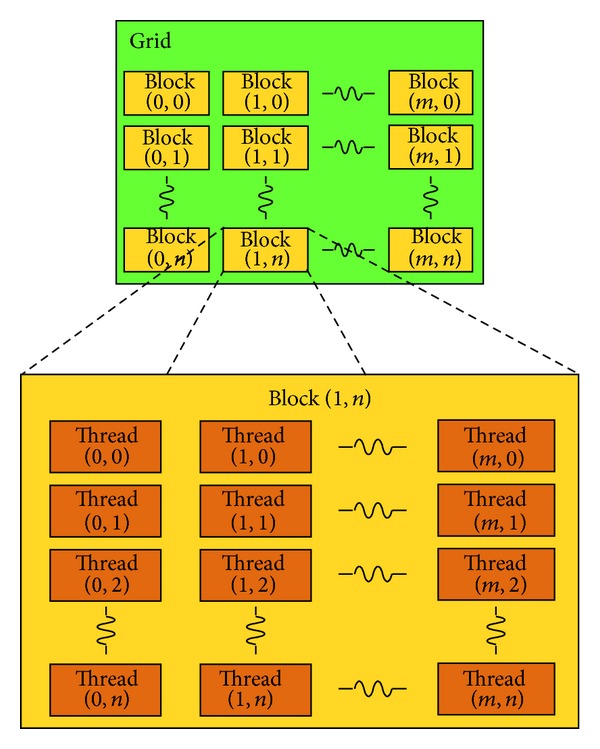
Grid structure of thread blocks.

As shown in Figure 2, kernel function is organized in the form of grid. The grid is composed of a certain number of blocks, and each block can be further partitioned into many threads. It is exactly this kind of structure that makes the two levels of parallel in kernel: parallel execution of all blocks in a grid and parallel running of all threads in a block. This is one of the most significant innovations of CUDA compared with traditional GPGPU programmatic interfaces. Each thread in kernel has its own block ID and thread ID to be distinguished with other threads. The other great innovation of CUDA is the realization of communication among different threads in the same block, mainly through shared memory and synchronization. 

### 2.2. Traditional NL-Means for 3D Images

Unlike local methods, the NLM filter does not make any assumptions about the location of the most relevant pixels used to denoise the current pixel. It explores image self-similarities by comparison of image patches and uses the weighted average of all the pixels in the image for noise reduction. For a 3D volume, the gray level NL(*v*)(*i*) of voxel *i* restored by the traditional NLM algorithm is the weighted average of gray scale of all voxels in the volume data *I*; that is

(1) $$\begin{array}{c} \text{NL}\left(v\right)\left(i\right)=\underset{j\in I}{\sum }w\left(i,j\right)v\left(j\right), \end{array}$$

where *v*(*j*) is the gray level of voxel *j* and *w*(*i*, *j*) is the weight given to *v*(*j*) in the calculation of voxel *i*, reflecting the similarity of voxels *i* and *j*. The weight depends on the local neighborhoods *N*_*i*_ and *N*_*j*_ (i.e., the similarity window) centered at the voxels *i* and *j* and it is computed as

(2) $$\begin{array}{c} w\left(i,j\right)=\frac{1}{Z\left(i\right)}e^{-({||v(N_{i})-v(N_{j})||}_{2,\alpha }^{2}/h^{2})}, \end{array}$$

where *h* acts as a smoothing parameter controlling the decay of the exponential function, *Z*(*i*) is a normalization constant with *Z*(*i*) = ∑_*j*∈*I*_ *e* ^−(||*v*(*N*_*i*_)−*v*(*N*_*j*_)||_2,*α*_^2^/*h*^2^)^ to ensure ∑_*j*∈*I*_ *w*(*i*, *j*) = 1, and ||·||_2,*α*_^2^ is the convolution for the Euclidean distance and the Gaussian kernel with the standard deviation *α*. 

For practical computational reasons, the number of voxels taken into account in the weighted average is usually limited to the so-called search window, which also centers at voxel *i*. The bigger the search window, the better the denoising effect, but the longer the processing time.

### 2.3. Bayesian Theory Based 3D NL-Means

Since the traditional NLM filter is originally designed for Gaussian noise removal, it cannot be directly applied to denoising ultrasonic images corrupted with the speckle noise. Recent studies related to US images demonstrated that the distribution of noise can be satisfyingly approximated by a Gamma distribution [20]. So, the following noise model was used in this paper [7]:

(3) $$\begin{array}{c} u\left(x\right)=v\left(x\right)+v^{\gamma }\left(x\right)\eta \left(x\right), \end{array}$$

where *v*(*x*) is the original image, *u*(*x*) is the observed image, *η*(*x*) ~ *N*(0, *σ* ^2^) is a zero-mean Gaussian noise with variance of *σ* ^2^. It has been shown in [21] that an empirical estimator of an image patch *B*_*i*_ can be defined as

(4) $$\begin{array}{c} \overset{\wedge }{v}\left(B_{i}\right)=\frac{\left(1/\Delta_{i}\right)\sum_{j=1}^{\left|\Delta_{i}\right|}v\left(B_{j}\right)p\left(u\left(B_{i}\right)\;|\;v\left(B_{j}\right)\right)}{\left(1/\Delta_{i}\right)\sum_{j=1}^{\left|\Delta_{i}\right|}p\left(u\left(B_{i}\right)\;|\;v\left(B_{j}\right)\right)}, \end{array}$$

where *p*(*u*(*B*_*i*_) | *v*(*B*_*j*_)) denotes the probability density function of *u*(*B*_*i*_) given the noise free and unknown patch *v*(*B*_*j*_) and Δ_*i*_ stands for the search window centered at voxel *i* of size |Δ_*i*_|. Since *v*(*B*_*j*_) is unknown, an estimator is classically computed by substituting *u*(*B*_*j*_) for *v*(*B*_*j*_). Assuming that *u*(*x*) | *v*(*x*) ~ *N*(*v*(*x*), *v*(*x*)^2*γ*^ *σ* ^2^) for the Gamma distribution noise model, we can express the probability density function as

(5) $$\begin{array}{c} p\left(u\left(x\right)\;|\;v\left(x\right)\right)\propto \exp-\frac{{\left(u\left(x\right)-v\left(x\right)\right)}^{2}}{2v{\left(x\right)}^{2\gamma }\sigma^{2}}. \end{array}$$

Given a block *B*_*i*_ (i.e., the reference patch), the distance for calculating the similarity between volume patches can be defined as

(6) $$\begin{array}{c} dp\left(u\left(B_{i}\right),u\left(B_{j}\right)\right)=\overset{P}{\underset{p=1}{\sum }}\frac{{\left(u^{\left(p\right)}\left(B_{i}\right)-u^{\left(p\right)}\left(B_{j}\right)\right)}^{2}}{{\left(u^{\left(p\right)}\right)}^{2\gamma }\left(B_{j}\right)}, \end{array}$$

where *P* denotes the size of *B*_*i*_. Loupas et al. have shown that *γ* = 0.5 model fits better to data than the multiplicative model or the Rayleigh model based on the experimental estimation of the mean versus the standard deviation in Log-compressed images [22]. By setting *γ* = 0.5, we can derive the weight from (1) and (6) as

(7) $$\begin{array}{c} w\left(B_{i},B_{j}\right)=\frac{1}{Z_{i}}\exp\left(-\frac{1}{h^{2}}\overset{P}{\underset{p=1}{\sum }}\frac{{\left(u^{\left(p\right)}\left(B_{i}\right)-u^{\left(p\right)}\left(B_{j}\right)\right)}^{2}}{u^{\left(p\right)}\left(B_{j}\right)}\right). \end{array}$$

### 2.4. Block-Wise Optimization

A block-wise implementation of the proposed NLM-based filter can significantly reduce the computational burden while maintaining excellent restoration quality [23] only by taking the similarity of two voxels in the voxel-wise method in formula (7) as the similarity of two blocks in the block-wise method. Here, we briefly describe the steps of carrying out the block-wise NLM algorithm for 3D ultrasound images.

(1) Divide the original 3D volume *Ω* ^3^ into overlapping blocks *B*_*i*_ of size *P* = (2*R* + 1)^3^; that is, *Ω* ^3^ = ∪_*i*_ *B*_*i*_. These patches are centered at different voxels which constitute a subset of *Ω* ^3^ and the distance between the centers (i.e., the step size) of two neighboring reference patches is set to be *n*.
(2) Set the size of the search window to be (2*M* + 1)^3^. Then, the similarities between the reference patch and all similarity windows in the corresponding search window will be obtained by formula (7). So, a reference patch *B*_*i*_ can be restored as follows:
  (8) $$\begin{array}{c} \text{NL}\left(u\right)\left(B_{i}\right)=\underset{B_{j}\in \Delta_{i}}{\sum }w\left(B_{i},B_{j}\right)u\left(B_{j}\right). \end{array}$$
(3) For a voxel *x*_*i*_ included in several blocks *B*_*i*_, several estimations of the same voxel *x*_*i*_ from different NL(*u*)(*B*_*i*_) are computed and stored in a vector *A*_*i*_. The final restored intensity of voxel *x*_*i*_ is the mean of all the restored values of voxel *x*_*i*_ in different blocks NL(*u*)(*B*_*i*_).

Indeed, for a volume *Ω* ^3^ of size *N* ^3^, the global complexity is *O*((2*R* + 1)^3^(2*M* + 1)^3^((*N* − *n*)/*n*)^3^). For instance, with *n* = 2, the complexity is divided by a factor 8 compared with the voxel-wise denoising algorithm. 

### 2.5. CUDA Accelerated 3D Block-Wise NL-Means

According to the principle of 3D block-wise NLM, the restoration task of each reference patch is very suitable for GPU implementation since each patch can be denoised independently. Then, we can use kernel function for image denoising. 

Firstly, the proper size of the reference patch, the search window, and the step size should be choosen. Then, we need to read the 3D volume data to CPU and do some initialization for CUDA, and subsequently allocate device memory for preparation of data transfer to GPU. Let the half-length of the reference patch, the search window, and the step size be *Ref_R*, *Sch_R*, and *Stp_S*, respectively. The size of the input data, *insize*, and the size of the output data, *outsize*, of the kernel function should be

 
*unsigned int insize = W∗H∗F; //original data size *
 
*int NUM_BX= (W-2∗Sch_R-Ref_L)/Stp_S+1; //the number of reference patches in X axis *
 
*int NUM_BY= (H-2∗Sch_R-Ref_L)/Stp_S+1; //the number of reference patches in Y axis *
 
*int NUM_BZ= (F-2∗SCH_R-Ref_L)/Stp_S+1; //the number of reference patches in Z axis *
 
*unsigned int outsize = Ref_L ∗ Ref_L ∗ Ref_L ∗NUM_BX∗NUM_BY∗NUM_BZ;*

and *W*, *H*, and *F* represent the width, height, and depth of the original volume and *Ref*_*L* = (2*Ref*_*R* + 1). Since the format of all the data is float, we will allocate the device memory as follows:

 
*float∗d_idata; //kernel input *
 
*CUDA_SAFE_CALL(cudaMalloc((void∗∗)&d_idata,insize∗sizeof(float))); *
 
*float∗d_odata; //kernel output *
 
*CUDA_SAFE_CALL(cudaMalloc((void∗∗)&d_odata,outsize∗sizeof(float))).*

To hand the volume patch restoring task over to GPU, we need to determine two of the most crucial parameters affecting the whole computational time—the block size and the grid size. By running the Device Query program from CUDA SDK, we can achieve the maximum number of threads per block: 1024, and the maximum sizes of each dimension of a grid: 2147483647∗65535∗65535 (this is usually enough for practical ultrasound data). The multiprocessor creates, manages, schedules, and executes threads in groups of 32 parallel threads called warps. When a multiprocessor is given one or more thread blocks to execute, it partitions them into warps; and each warp gets scheduled by a warp scheduler for execution. A warp executes one common instruction at a time, so full efficiency is realized when all 32 threads of a warp agree on their execution path. To get higher efficiency of the multiprocessor, the number of threads per block should be a multiple of 32. According to our experiments, the fastest results were achieved by partitioning each block into 64 threads. So our block and grid are organized like this:

 
*dim3 threads(64,1,1); *
 
*int N_X = (NUM_BX+63)/64; *
 
*int N_YZ = NUM_BY∗NUM_BZ; *
 
*dim3 grid(N_X,N_YZ,1); *
 
*NLM_kernel<<<grid,threads>>>( ).*

Here, *NLM_kernel* is the kernel function used to denoise all the reference patches. Each block and thread in the grid can be identified by a one-dimensional, two-dimensional, or three-dimensional index accessible within the kernel through the built-in *blockIdx* and *threadIdx* variables. Thus, we can get the exact position of each voxel in kernel function:

 
*const int bx = blockDim.x∗blockIdx.x+threadIdx.x; *
 
*const int bz = (blockDim.y∗blockIdx.y+threadIdx.y)/NY; *
 
*const int by = (blockDim.y∗blockIdx.y+threadIdx.y)-bz∗NY.*

Here, *NY* denotes the number of overlapping blocks in *Y* axis, which is equal to *NUM_BY* when the kernel function is called. Within kernel function, we need to do an outer cycle process and an inner cycle process. The inner cycle process is to calculate the distance for similarity between the reference window and the similarity window, and the outer cycle process aims at calculating the similarity between the reference window and all the similarity windows in the corresponding search window, thus obtaining the restored result of the reference window. Here is the pseudocode of the Algorithm 1.

**Figure 3:**
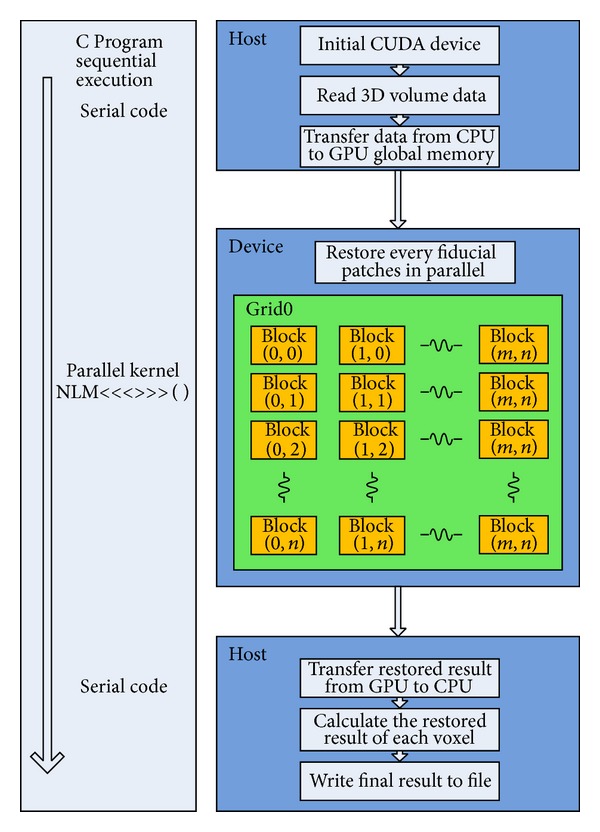
The flow chart of the implementation.

Through the processing of the kernel function, we will get a new volume composed of all the restored reference patches, so the next step is to calculate the restored result of each voxel by taking the average of the same voxel from different reference blocks. As we have partitioned the original volume into overlapping parts, one voxel can appear in a few different reference patches. So, the restoring task of each voxel is not a parallel processing. Besides, we need to do some judgment of the position of the voxels to determine the times they have been calculated. GPU is unfit for such problems. Hence, we put this computing part to CPU. The whole implementation of CPU and GPU cooperation is shown in Figure 3.

## 3. Result and Discussion

**Figure 4:**
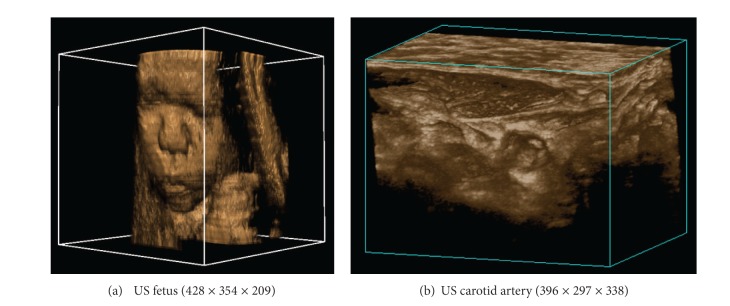
Real ultrasound volumes for experiment.

Experiments were made on real ultrasound volume data as shown in Figure 4, (a) is an US fetus with the size of 428 × 354 × 209, (b) is the US carotid artery with the size of 396 × 297 × 338. The operating system used was Windows 7 32-bit. The used CPU was an Intel(R) Core(TM) i3-2120 3.3 GHz with 4 processor cores, and the used GPU was a NVIDIA GeForce GTX 660 Ti, equipped with 1344 processor cores and 2 GB of memory. 

**Figure 5:**
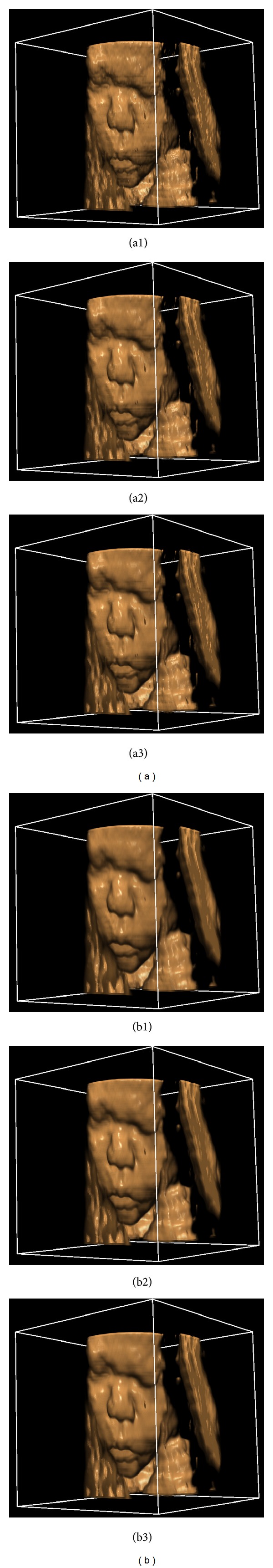
Denoised results of the ultrasound fetus: (a1)–(a3) the denoised result of the traditional NLM with the similarity window radius changing from 1 to 3; (b1)–(b3) the denoised result of the proposed NLM with the similarity window radius changing from 1 to 3.

In order to testify the performance of the proposed algorithm over to the traditional NLM, comparisons were made on the US fetus data as it involves more detail information. The decay parameter was set to a constant 20.0, and the distance of each reference block *Stp_S* was set to (*Ref*_*R* + 1) to ensure the edge continuity while controlling computational load. The similarity window radius was set from 1 to 3, and the size of search window was kept 11 × 11 × 11. As shown in Figure 5, the performance of our method is obviously better than that of the traditional NLM. The proposed method can acquire better denoising results while maintaining the image detail information.

**Figure 6:**
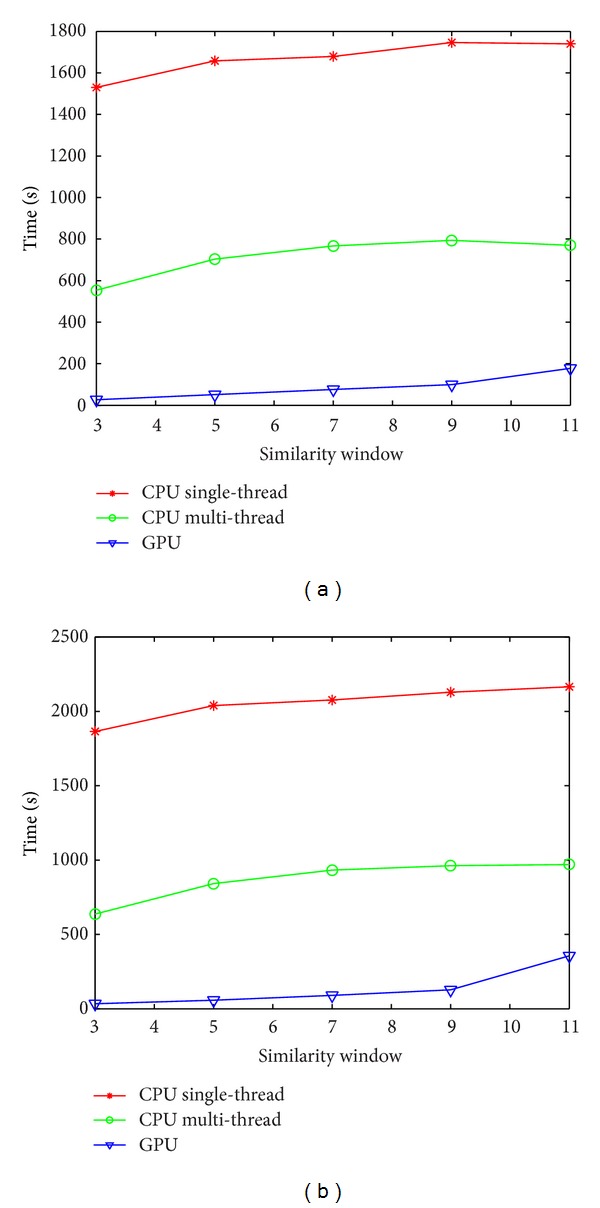
The performance comparisons of the proposed NLM implemented by single-thread CPU, multithread CPU, and GPU for the US fetus on the left and the US carotid artery on the right.

In order to evaluate the effect of the GPU acceleration, time comparisons of CPU single thread, CPU multithread, and GPU implementation of different parameters were made for the proposed method. The decay parameter was set to a constant 20.0. The size of search window was kept 11 × 11 × 11, and the distance of each reference block *Stp_S* was also set to (*Ref*_*R* + 1). Tables 1 and 2 show the processing time of CPU single thread, CPU multithread and GPU operations of the similarity window radius ranging from 1 to 5. The results of the experiments can lead us to the conclusion that the GPU acceleration can enormously improve the processing speed of the proposed NLM algorithm, for example, up to 57 times over the single thread CPU for the fetus data with the similarity window size of 3 × 3 × 3. While multithread of the CPU can accelerate the computation only to some extent. As we can see in Figure 6, the time of C++ implementation does not vary much with the increasing similarity window size. When the similarity window becomes bigger, the denoising calculation of each reference window becomes more complex while the total number of reference patches reduces because *Stp_S* is getting larger. So, the whole complexity does not change much. The observation from the GPU columns on the two tables shows that the processing time increases relatively faster with the increasing similarity window size compared to that of CPU, and accordingly the speed-up ratio decreases with the increasing similarity window size. The reason can be explained in this way. As we have mentioned above, the CUDA performance will be better when the arithmetic intensity is higher. It is easy to understand that the increasing similarity window size will lead to larger *Stp_S* and fewer reference patches, thus resulting in lower arithmetic intensity since the kernel function here serves for the restoring of each reference patch.

**Figure 7:**
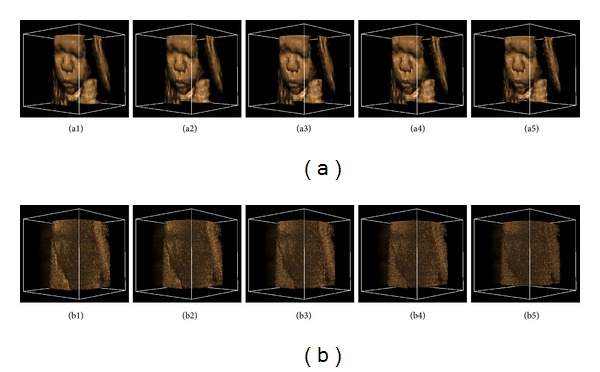
Denoised results of the ultrasound fetus: (a1)–(a5) the denoised result with the similarity window radius changing from 1 to 5; (b1)–(b5) the corresponding residual images between the original image and filtered images.

**Figure 8:**
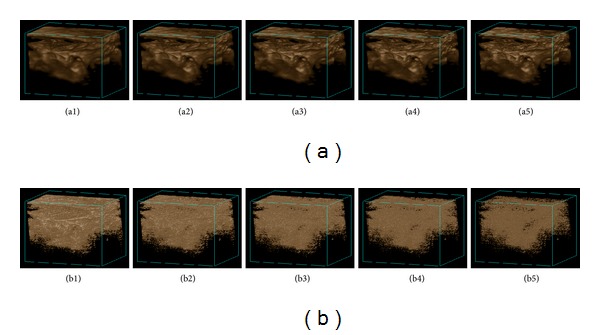
Denoised results of the ultrasound carotid artery. (a1)–(a5) the denoised result with the similarity window radius changing from 1 to 5; (b1)–(b5) the corresponding residual images between original image and filtered images.

We also give the denoised results using the different similarity window sizes ranging from 3∗3∗3 to 11∗11∗11 and the corresponding residual images between the original image and filtered images in Figures 7 and 8, from which we can see that the proposed method can effectively maintain the image structure details and at the same time remove the noises.

Though GPU can achieve very high efficiency for parallel computation because of its tremendous computational horsepower and very high memory bandwidth, generally it is not easy for programmers to accomplish the threads assignment work to the optimum efficiency. As the maximum number of threads in a block and the maximum number of blocks in a grid are predefined for a specific GPU, we cannot do the threads assignment work as we expect. Besides, the final efficiency depends on the computational intensity to some extent. As the GPU is very good at addressing such problems that can be expressed as data-parallel computations, the final performance will be better when the arithmetic intensity is higher. If the arithmetic intensity is low and involves many branches or judgments, the use of GPU may not be preferable.

## 4. Conclusion

In this paper, a GPU-based fast block-wise NLM algorithm for 3D ultrasound image was presented. While the high-performance computer clusters can improve the processing speed very much, the cost is usually too high for most researchers and clinical users. Though improving frequency and upgrading manufacturing process of CPU can enhance the computational speed for the algorithms of high time complexity such as NLM denoising, it is still difficult to meet the practical application requirement. As the algorithm computation is very dense and quite fit for parallel computation, GPU-based approach is introduced to accelerate the process by exploiting its powerful parallel computation abilities. Experiments on real ultrasound volume data showed that the proposed method is capable of enormously speeding up the NLM algorithm, and the speed-up ratio of the proposed method is better when the arithmetic intensity is higher. With an original volume data of a small size or a small search window chosen, the proposed method can be used for real-time despeckling of 3D US images. Future work will be focused on how to tune the decay parameter and the size of similarity window adaptively and extend the proposed method to 4D US video denoising, such as in echocardiography. We believe that the GPU-based Bayesian NLM method will be valuable in practical applications and GPU will be more widely used in the field of medical image processing. 

## Supplementary Material

This movie shows the visualization of the 3D ultrasound fetus, including the original image displaying and the denoised results by our algorithm. baby.428x354x209.raw is the original volume data. The CUDA_denoised_baby_Rx.raw is the denoised data by our algorithm with the reference window radius of x. The X, Y and Z sliders are used to control the clipping of the objects to remove the background. The line chart on the lower left corner is the transfer function adjusting module. The first display mode is the mode with pseudo color. The second display mode is the mode with both pseudo color and light. The third display mode is the mode of gray level.

## Figures and Tables

**Algorithm 1:**
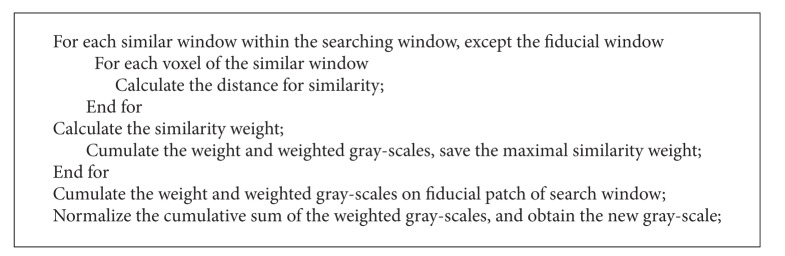


**Table 1:** Time comparisons of CPU single-thread, CPU multithread and GPU of ultrasound fetus denoising.

Similarity window	*Stp_S *	Processing time (s)		
		CPU single-thread	CPU multithread	GPU
3 × 3 × 3	2	1530.08	553.86	26.66
5 × 5 × 5	3	1657.64	703.13	51.15
7 × 7 × 7	4	1678.91	767.12	75.54
9 × 9 × 9	5	1745.82	793.68	99.11
11 × 11 × 11	6	1739.60	769.55	177.34

**Table 2:** Time comparisons of CPU single-thread, CPU multithread and GPU of ultrasound carotid artery denoising.

Similarity window	*Stp_S *	Processing time (s)		
		CPU single-thread	CPU multithread	GPU
3 × 3 × 3	2	1865.31	637.23	33.58
5 × 5 × 5	3	2039.22	841.77	57.22
7 × 7 × 7	4	2076.39	933.12	90.64
9 × 9 × 9	5	2129.09	962.11	127.96
11 × 11 × 11	6	2165.79	970.43	356.19
